# Supplementary material for: Prevalence and determinants of severity levels of anemia among children aged 6–59 months in sub-Saharan Africa: A multilevel ordinal logistic regression analysis
Source: PLoS One. 2021 Apr 23;16(4):e0249978. doi: 10.1371/journal.pone.0249978 (PMC8064743; doi:10.1371/journal.pone.0249978)
Supplement: Attachment — Submitted filename: Manuscript Number PONE-D-20-37075.pdf [file pone.0249978.s001.pdf]

Anaemia has been a public health burden among under-5 children in sub-Saharan African countries. Therefore, in trying to understand the prevalence and determinants of severity levels of anaemia among children aged 6-59 months in sub-Saharan Africa and ways of reducing the burden, I found merit in the paper.

The paper is well written, the issues tackled are relevant, the methodology applied is appropriate, and the results are properly written. However, the authors were not consistent with using the words anaemia and anemia, sub-Sharan, and Sub-Saharan. Therefore, the entire manuscript requires thorough proofreading and editing.

In lines 51, 66, 67, 68, 69, 71, 75, 78, and 79 of pages 3-4, anemia should be changed to anaemia. Please be consistent and edit the entire paper.

In lines 70, 86, and 92 of page 3, sub-Saharan Africa should be written as sub-Saharan Africa. Please check the entire paper.

Methods

In line 103 of page 4, refer the readers to Table 1 that shows the list of countries involved in the study. In lines 104 and 106, change Sub to sub. There was no elaboration on the study population and sample size. The inclusion and exclusion criteria were also missing. Highlight information got from the DHS. The paper should also explain how the datasets from the surveys were merged.

The authors failed to list the percentage distribution of anaemia in each of the 32 countries.

In line 23 of page 2, the authors wrote anaemia and later changed it to anemia in line 26 of page 2. Hence, they continued interchanging the two words. Thorough editing is highly required.

The -2LLN acronym appeared first in line 179 of page 8; therefore, the authors should write the full meaning at the location in the article.

On page 7, the authors should clearly explain the analytical strategy and process, providing references.

Results and discussion

In line 194 of page 9, the authors mention the percentage distribution of anaemia among children in East Africa. It is also necessary that the percentage distribution of childhood anaemia in West Africa, Central Africa, and Southern Africa should be mentioned.

In line 202 of page 10, change Sub to sub.

In lines 217, 219, 221, 222, and 227 of page 10, change anemia to anaemia. Please be consistent with one and edit the whole manuscript.

In line 272 of page 12, South Africa should be changed to Southern Africa.

Correct the anemia in line 279 and Sub-Sharan Africa in line 280 of page 12.

A table of unadjusted ORs is missing.

Lines 287, 290, 291, 296, and 307 of page 13 should also be corrected.

Correct the anemia in lines 326 and 327 of page 14.

Lines 334 and 335 of page 15 should also be corrected.

Correct the anemia in lines 372 and 381 of page 16.
