# Supplementary material for: Prevalence and determinants of severity levels of anemia among children aged 6–59 months in sub-Saharan Africa: A multilevel ordinal logistic regression analysis
Source: PLoS One. 2021 Apr 23;16(4):e0249978. doi: 10.1371/journal.pone.0249978 (PMC8064743; doi:10.1371/journal.pone.0249978)
Supplement: Attachment — Submitted filename: Point by point response.docx [file pone.0249978.s003.docx]

**Point by point response for editors/reviewers comments**

**PLOS ONE Journal**

**Manuscript title:** Prevalence and determinants of severity levels of anemia among children aged 6-59 months in sub-Saharan Africa: a multilevel ordinal logistic regression analysis

**Manuscript ID:**  PONE-D-20-37075R1

**Dear editor.**

Dear all,

We would like to thank you for these constructive, building, and improvable comments on this manuscript that would improve the substance and content of the manuscript. We considered each comment and clarification questions of editors and reviewers on the manuscript thoroughly. Our point-by-point responses for each comment and question are described in detail on the following pages. Further, the details of changes were shown by track changes in the supplementary document attached.

**Response to Reviewer comment**

**Reviewer#3**

1. - Point 8. The main article:

In background section:

Reviewer comment: In page 3, lines 49-50; “... World Health Organization (WHO) defines childhood anemia as a Hb concentration below 110 g/L.” The WHO has three cut-off levels for defining anaemia in childhood depending on age group. Please revise and correct your statement. Please revise.

Authors’ response: Thank you for the comments. We corrected the references. See the revised manuscript

Reviewer feedback 1: childhood do not denote for under five years old children. As mentioned above this cut-off level of haemoglobin is not valid for all childhood age categories. It is only valid for children under the age of 5 years. Author has corrected the reference but not the sentence. Kindly, correct your statement about this. Moreover, this reference used from the WHO is less relevant. It is about iron deficiency anaemia. The WHO has other resources which tell exactly what is the different cut-off levels for defining anaemia in different age, population, and physiological status. Furthermore, the reference used was not properly cited.

**Authors’ response**: Thank you for the comment. We accept the comment and rewrite as anemia among under-five children. (See the revised manuscript)

2. Previous reviewer comment

Point 13. In page 4, lines 90-92; the author justified for this study the cause of “very few studied conducted” on anaemia; while he is using data from 32 studies in the region from the DHS only. This fact is contradicting the author statement. Furthermore, and since the author did not conduct an original research to fill the mentioned gab in knowledge, but only analyzing what have already done, then the limited number of publications could not be considered as an adequate justification for this research. Consider revising the gap in knowledge and restatement of the study justification.

Authors’ response: Thank you for the comments. As we stated in the background section, we justify the significance of the study from public health perspective and methodological perspective. Regarding the public health perspective, this study was based on the pooled DHS data of 32 sub-Saharan African countries with a very large sample size and this could increase the power of the study and the estimate can be generalized. Besides, the use of multilevel approach, is mainly concentrated on the ecological approach of epidemiology as it can take into account the neighborhood effect, and the result can give the overall picture of SSA. Regarding the methodological perspective, as you can see previously published literatures treat anemia as a binary outcome by categorizing no/yes but as you can understand treating mild, moderate, and severe anemia as yes is not statistically appropriate since there is the loss of information because the factor responsible for mild anemia may not be similar with the factor that can cause severe anemia. Therefore, we applied the multilevel ordinal logistic regression model to get a reliable estimate and avoid loss of information. (See the Background section).

Reviewer feedback 2: these justifications are acceptable. But the statement of “very few studied conducted” is not acceptable. This undermines others’ work. In fact, there are many studies on anaemia prevalence and its determinants available as articles and other resource literature. You can include what you have stated above in your article, but you need to revises the phrase about availability of “very few studies on anaemia” as a justification.

**Authors’ response**: Thank you for the comments. We accept the suggestions and modified the revised manuscript. (See the revised manuscript)

3. Point 14. Methods:

In page 5, line 113; how were households been selected? In page 5, line 114; how were children selected within households?

Authors’ response: Thank you for the comments. …. We reported the DHS databases to link for further methodological procedures. In the selected households the most recent children were selected for this study. (See the Method section)

Reviewer feedback 3: In your study, you need to describe the methodology clearly and in details. Sample selection method should receive special concern and detailed description to ensure that the study design is properly fit the data and its collection method. Yes, you could refer readers to the website/other reference for more details, but the details that describe your study. In your case, describing selection of the sample at each level has its implication on choosing the multilevel model for your analysis and the way that you considered the levels. More over describing the methodology will give an indication about the extent to which methods are homogenous in all countries and that they could be used in your study without the need for prior manipulation/re-arrangement. If children were not randomly selected then how can you explain avoiding bias in selection.

So, you need to describe how households were selected. The same apply for child selection with each household.

**Authors’ response**: Thank you for the comments. We included the statement about how the households selected under the method section of the manuscript. (See the revised manuscript)

4. Point 18. In page 6, lines 128-129; … How was the distance to health facility assessed? How was the size of the child being assessed and categories defined?

Authors’ response: Thank you for the comments. …. Regarding health distance facility was assessed subjectively asking a question how do you see the distance to reach health facility and they responded as a big problem and not a big problem. Whereas, about the size of the child at birth, it was assessed by asking mothers what was the size of the child at birth and they responded as very small, small, average, large, and very large.

Reviewer feedback 4: The author needs to mention these explanations the article to clarify these issues. However, in response to point 29 in the discussion section, this subjective measurement of size of the baby creates incomparable results with what is available in the literature as stated by the author! So, the author may think of removing variables with non-specific definitions/measurement.

Related to this is that, “distance to health facility” was removed from the text while it was still there in table 4. However, it is not mentioned in tables 2 and 3! Kindly, assure consistency and cohesion.

More issues related to this include mismatch between what is in the text as variables and what is in table 2 and 3. PNC visit was mentioned in table 2 where it is not there in the article. For table 3, many important variables disappeared from table 3 while they were mentioned in table 2. These includes source of drinking water, sex of the head of the household, wanted pregnancy, employment status of the mother, smoking status of the mother, cough, receiving antiparasitic drugs and vit. A supplementation.

**Authors’ response**: Thank you for the comments. regrading distance to health facility, we have removed from the manuscript as it is more of subjective, and we included PNC in the variable of the study section but was not included in the model as it has p-value>0.2 in the bi-variable analysis. Regrading birth size of a child, as you know birth weight of the child was assessed in two ways such as mothers perceived birth size of a child and measured birth weight of the child but the measured weight were missed in more than 80 percent of child’s. So, in developing countries mothers perceived size is commonly used and it has more than 90 percent overall agreement with the measured Birth weight. Besides, we acknowledge in the limitation section.

For Table 3, we presented the severity level of anemia by selecting the commonly reported predictors of anemia that is why we missed some of the variables in Table three.
